# Analysis of the Impact of Invisible Road Icing on Selected Parameters of a Minibus Vehicle

**DOI:** 10.3390/s22249726

**Published:** 2022-12-12

**Authors:** Dariusz Kurczyński, Andrzej Zuska

**Affiliations:** Department of Automotive Engineering and Transportation, Faculty of Mechatronics and Mechanical Engineering, Kielce University of Technology, al. Tysiąclecia Państwa Polskiego 7, 25-314 Kielce, Poland

**Keywords:** traffic safety, vehicle movement sensors, critical traffic conditions, vehicle dynamics, vehicle braking, vehicle acceleration, circular driving, road icing

## Abstract

The measurement of acceleration during vehicle motion can be used to assess the driving styles and behaviours of drivers, to control vehicle traffic, to detect uncontrolled vehicle behaviour, and to prevent accidents. The measurement of acceleration during vehicle motion on an icy road can be used to warn the driver about changing conditions and the related hazards. This paper presents the results of testing the motion parameters of a Ford Transit adapted for passenger transport in critical traffic conditions. It can contribute to the improvement of road safety. Critical traffic conditions are deemed in the paper as sudden braking, rapid acceleration, and circular vehicle motion at maximum speed maintainable in the given conditions. The vehicle’s acceleration and speed were measured during the tests. The tests were carried out with a TAA linear acceleration sensor and a Correvit S-350 Aqua optoelectronic sensor. The same test runs were conducted on a dry surface, a wet (after rain) surface and a surface covered with a thin, invisible ice layer. The objective of the tests was to determine the impact of invisible road icing, the so-called black ice, on the tested vehicle’s braking, acceleration, and circular motion. It was demonstrated that a virtually invisible ice layer covering the road surface has a substantial impact on the tested vehicle’s motion parameters, thereby affecting traffic safety. It substantially extends the braking and acceleration distances and requires the driver to reduce the vehicle’s speed when performing circular motions. A clear wet surface, representing motion after rain, did not substantially affect the analysed parameters. The obtained results can be used in traffic simulations and to analyse the causes of accidents.

## 1. Introduction

### 1.1. Motivation

The impact of weather conditions on road traffic safety is very large. Special traffic disruptions are caused by freezing rain, ice, snow, heavy rain, fog, and haze. Extreme weather phenomena are currently intensifying due to global warming. They can contribute to road accidents and affect the functioning of transport systems. Unfavourable weather conditions impede driving as they reduce visibility, lower the grip, and affect the drivers’ condition as well as physical and mental abilities. This leads to an increased probability of adverse events. Road accidents and transport issues generate high human, medical, ecological, economic, and property costs. This makes road safety problems a very important area of research interest [1,2,3,4,5,6]. One of the ways to improve it is to install sensors in cars, which will allow us to measure the acceleration values acting on the vehicle [7,8,9,10]. These values can then be used to evaluate vehicle movement, including traffic conditions and driver behaviour. An important parameter for safety is the braking distance and the factors that have a significant impact on it [11,12,13].

The impact of weather conditions on transport was the subject of various studies and analysis [14,15,16,17,18,19]. Weather conditions affect road and environment properties, vehicle properties, and driver predisposition and abilities. It is difficult and ambiguous to assess the impact of weather conditions on road traffic safety. It depends on many factors that depend on one another and their contribution to possible accidents is difficult to determine. It often utilizes empirical studies to assess the relationships between weather conditions and the possibility of accidents [20,21,22,23,24,25].

The joint effect of weather and lighting conditions on injury severity in single-vehicle accidents was investigated in paper [26]. The severity of injuries to road accident participants in winter conditions was investigated in paper [27]. Research presented in paper [28] showed that injury severity in single-vehicle crashes was related to wind speed, rain, humidity, and air temperature. The authors of paper [29] showed that weather conditions have a significant impact on highway traffic safety. Paper [30] investigated the effects of age, gender, and road conditions on accident severity. Accidents on dry, wet, and snow/ice-covered carriageway surfaces were analysed. In paper [31], the effects of rain and fog on traffic parameters were studied using weather and traffic sensors. The authors of this work used an acceleration sensor and a speed sensor to investigate the effect of an invisible thin layer of ice (black ice) on the traffic parameters of a selected minibus vehicle.

### 1.2. Analysis of Research Presented in the Literature

Black and Mote analysed the relationships between winter precipitation and the possibility of an accident [14]. They demonstrated that winter precipitation causes an increase in road accidents and injuries when compared to traffic conditions with no precipitation. They also demonstrated that more severe precipitation caused a higher probability of accidents and injuries when compared to less severe precipitation. Andrey et al. demonstrated that snowy, freezing rain days, or days with other freezing precipitation, feature higher collision numbers [15]. There are more collisions on roads with higher maximum traffic speeds. Malin et al. demonstrated that the risk related to bad weather and traffic conditions was higher on motorways when compared to other road types [22]. Eisenberg and Warner demonstrated that the first snowy day in a given year was much more dangerous than other snowy days in terms of fatalities [17]. Fog and haze on roads with high traffic intensity and high driving speeds cause serious road accidents with a large number of vehicles involved. In their studies, Mueller and Trick demonstrated that fog causes a reduced view distance and that the distance to the vehicle in front is misjudged [32]. This causes road accidents.

Based on their research, Carson and Mannering state that the use of ice warning signs on roads did not substantially contribute to the reduction of accidents and their effects [16]. In their paper, Andrey et al. demonstrate that drivers react insufficiently to changing weather conditions related to snow or freezing rain [15]. Such precipitation causes substantial changes to vehicle motion conditions. Aside from limited visibility, there may be a change in grip. In their research, Cheng at al. demonstrated that the tyre grip coefficient on roads covered with snow is between 0.18–0.31, while on mixed icy and snowy roads is between 0.06–0.17, and it decreases along with an increase in air humidity [33]. For comparison, the tyre grip coefficient on dry asphalt surfaces is within the range of 0.7–0.9 [34]. Malin et al. demonstrated that a higher accident risk was obtained for freezing rain, and for slippery and very slippery road conditions [22].

Weather conditions shape the road traffic conditions. They strongly affect the road surface condition, visibility, and the operation of specific vehicle mechanisms. Vehicle motion parameters that affect safety change depending on the road surface condition. Surface condition affects the vehicle’s braking distance, acceleration, and lateral acceleration, effecting the vehicle during curvilinear motion. The aforementioned quantities are subjects of road studies and analyses. They are taken into consideration when analysing the causes of road accidents and traffic simulations. Jang proposed the possibility of road slipperiness detection based on wheel skid and wheel acceleration [35]. He pointed out that this can be done by using the data obtained from the sensors of a digital tachograph (DTG) which is an obligatory device for commercial vehicles. Ogura et al. proposed and tested a road surface detection system with the objective to build a tool intended to identify the road surface grip coefficient [36]. Koglbauer et al. tested the enhancement of capabilities of the autonomous emergency braking (AEB) system by introducing the system’s adaptation to the changing friction between the tyre and the road, e.g., on a snowy road [37]. This can be a step towards further road traffic safety improvement, especially in winter conditions.

Koylu and Tural took on the issue of steering and braking in vehicles in ABS at low starting speeds in critical road conditions (slippery and wet surfaces) [38]. Low initial braking speed during ABS-assisted braking causes a pressure build-up and reduction in the braking system over a longer time period. Koylu and Tural demonstrated that a low speed impairs braking stability, control, and effectiveness during ABS-assisted braking, regardless of road conditions. They stated that weak wheel speed signals, high slipperiness dynamics, long-term braking pressure delays caused by pumping losses, and insufficient valve reaction time, substantially impede braking stability and effectiveness due to low ABS control efficiency. They proposed changing the ABS control algorithm.

Tyre grip plays a substantial role due to the transmission of forces between the tyres and the road surface. It substantially affects traffic safety. Kordani et al. conducted simulation tests of the impact of changes in the grip coefficient, depending on weather conditions, on braking [39]. The testing was conducted for three vehicle models: a sedan, a commercial vehicle, and a bus. It was demonstrated that grip coefficients of 0.9, 0.8, 0.7, and 0.6 achieved in the simulation show no substantial differences in braking distance. These values can be related to traffic conditions without atmospheric precipitation. On the other hand, the authors attributed the grip coefficients of 0.5, 0.4, 0.28, and 0.18 to the following weather conditions: wet, rainy, snowy, and icy surface. Substantial differences in the braking distance were obtained for these values. In paper [34], the tyre grip on a wet asphalt surface was in the range of 0.5–0.7.

Papers [40,41] presented various results of testing the friction between rubber and snow. Klein-Paste and Sinha conducted testing of friction between rubber and ice, as well as between sand and ice [40]. The friction between rubber and ice was low near the melting point and increased along with the decreasing ice temperature. Friction decreased substantially in the presence of a very thin snow layer. The sand-ice friction depended on the ice temperature to a lesser degree, while the presence of snow was not as impactful as in the case of the rubber-ice friction. Ella et al. demonstrated that the friction coefficient for rubber on packed snow decreases along with an increase in speed due to the water layer formed as result of heating caused by the friction [41].

Szumska et al. tested the braking distances and deceleration of a passenger vehicle on various surfaces: dry, wet, and contaminated [42]. The tests were also conducted on mixed surfaces. The tests involved driving one side of the vehicle on dry asphalt and the other on asphalt covered with dry sand, wet sand, or wet asphalt covered with wet sand. It was demonstrated that braking on mixed surfaces is more dangerous. Mixed surfaces extend the braking distance and can lead to vehicle stability losses.

Waluś tested the acceleration and braking on fresh snow. The tests were conducted on a passenger vehicle equipped with winter tyres [43]. The tests were conducted on different days in various weather conditions. The average full deceleration amounted to 2.91 ÷ 3.33 m/s^2^, while acceleration was in the range of 1.08 ÷ 1.23 m/s^2^. It was pointed out that winter conditions feature a considerable dispersion of the obtained results even when using the same vehicle, tyres, test location, driver, and measurement instruments. Extensive research on driving a car on ice- and snow-covered roads was conducted by Cheng et al. [33]. They tested the friction coefficient, driver reaction time when braking, and the braking distance on various snowy and icy surfaces. They proposed a method to calculate the maximum safe speeds at various speeds and types of surfaces covered by ice and snow.

### 1.3. Research Contribution

Literature does not feature many papers that present the results of testing the impact of weather conditions on the vehicles’ traction properties. It was impossible to find a contemporary paper that presented results of testing the impact of black ice covering an asphalt surface on the tested vehicle’s motion parameters for braking, acceleration, and driving in a circle in critical conditions. Black ice is a thin frozen water layer on the road surface, which is difficult to see for the driver. The purpose of this paper was to determine the impact of a thin and invisible ice layer (black ice) on the braking, acceleration, and circular motion of a Ford Transit adapted for passenger transport. The obtained results can be a source of data for motion simulations of a selected vehicle type and accident cause analyses. Furthermore, they can be a warning for drivers to become aware of the risk of driving in unfavourable weather conditions without taking special care.

## 2. Materials and Methods

### 2.1. Testing Methodology

**Figure 1:**
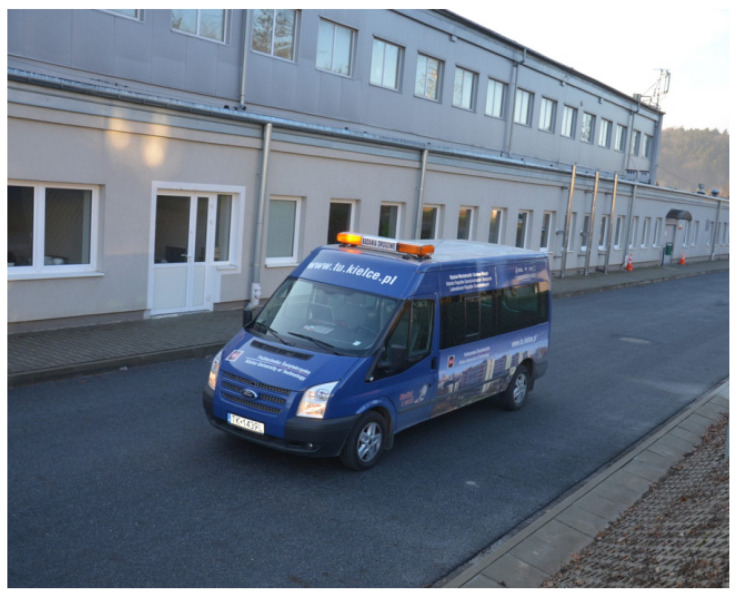
Ford Transit test vehicle.

The testing was conducted at the Automotive Engineering Laboratory of the Department of Automotive Engineering and Transport, at Kielce University of Technology. The tests were carried out using testing grounds with asphalt surfaces. The test subject was a sixth-generation Ford Transit adapted to transport nine passengers. The vehicle’s curb weight amounts to 2070 kg, whereas the laden mass amounts to 3050 kg. The vehicle used in the testing was equipped with a compression ignition engine, a cubic capacity of 2198 cm^3^, and rated power of 92 kW. Vehicles of this type are referred to as minibuses. The vehicle’s technical condition, especially the tyres and braking system elements, were checked prior to testing. The tested vehicle was equipped with summer Continental ContiVanContact 200, 205/65 R16C tyres with very little wear. The tread depth demonstrated wear of approximately 1 mm when compared to the nominal value. The tyre parameters of the Ford Transit test vehicle are presented in Table 1. The vehicle was not loaded during the tests. The vehicle’s load included the testing instrumentation and two people: the driver and other person who operated the testing instrumentation. The vehicle featured traction improvement systems (ABS, ASR, and ESP) which were enabled during testing. The view of the Ford Transit test vehicle is presented in Figure 1.

The testing was conducted in late autumn during sunny weather and at temperatures slightly above 0 °C. The testing was conducted on an asphalt surface in very good technical condition. The surface did not include any irregularities and defects. It is a non-public road, in a restricted area. The first test series was conducted on dry asphalt. The second test series was conducted on wet asphalt. The surface was covered in water prior to the testing. This represented the driving conditions after rain. The surface did not feature any puddles or standing water. In the latter part of this elaboration, this surface is referred to as wet asphalt (after rain). The third test series was conducted on the next day, at early morning hours, when the water froze on the asphalt, at a temperature of approximately −1 °C. The asphalt featured an invisible thin ice layer, the so-called “black ice”, which can be a significant surprise for the driver and a safety hazard.

Rapid (emergency) braking cycles, rapid acceleration cycles, and circular driving cycles were performed in each test series. Ten rapid braking cycles were performed for each surface condition. Each time, the driver accelerated the vehicle to approximately 50 km/h and then pressed the brake pedal sharply as in the case of an impending collision. The tests also featured rapid braking cycles from the starting speed of approximately 30 km/h. Ten rapid acceleration cycles were performed for each of the tested road surfaces. After stopping, the vehicle was accelerated to 50 km/h in the shortest time possible. The driver was tasked with accelerating the vehicle to the set speed by using the vehicle’s full capabilities and the tyre grip. The last cycle was driving in a circle with the highest possible fixed speed while maintaining the vehicle stability. The vehicle motion parameters when driving in a circle were tested in two directions. First, when the vehicle was driven counter clockwise (to the left). Then, the vehicle was driven clockwise in a circle (to the right).

The first test series’ measurements were used to develop plots for vehicle deceleration and speed during braking. The following parameters were designated based on an analysis of these plots: initial braking speed, V_o_; average initial braking speed for the cycles performed, V_om_; maximum recorded deceleration, a_max_; average maximum deceleration, a_maxm_; mean fully developed deceleration, MFDD; average mean fully developed deceleration, MFDD_m_; deceleration rise time, t_r_; average deceleration rise time, t_rm_; average fully developed deceleration time, t_MFDD_; and average mean fully developed deceleration time, t_MFDDm_. The average fully developed deceleration, MFDD (m/s^2^), and initial braking speed, V_o_ (km/h), were used to calculate the braking distance from the following relationship:

(1) $$S_{H}=\frac{V_{o}^{2}}{2\cdot MFDD}$$

The average braking distance, S_Hm_, was also designated for the performed rapid braking cycles.

The average mean fully developed deceleration, MFDD, was designated according to the formula presented in Regulation no. 13 of the UN’s Economic Committee for Europe [44]:

(2) $$MFDD=\frac{V_{b}^{2}-V_{c}^{2}}{25.92\cdot (S_{c}-S_{b})}$$

where V_b_ (km/h)—vehicle speed corresponding to 0.8 of the initial braking speed V_o_, V_c_ (km/h)—vehicle speed corresponding to 0.1 of the initial braking speed V_o_, S_c_ (m)—distance travelled between speed V_o_ and speed V_c_, and S_b_ (m)—distance travelled between speed V_o_ and speed V_b_.

The courses of the test vehicle’s measured longitudinal accelerations and speeds were developed and analysed for acceleration cycles up to 50 km/h. The following parameters were read based on the developed plots: maximum acceleration during acceleration cycles, a_rmax_; time between starting the vehicle and achieving maximum acceleration, t_armax_; time between starting the vehicle and achieving 50 km/h, t_50_; and distance travelled from the time of starting the vehicle to achieving 50 km/h, S_50_. The following parameters were also calculated: average maximum acceleration during acceleration cycles, a_rmaxm_; average time between starting the vehicle and achieving maximum acceleration, t_armaxm_; average time between starting the vehicle and achieving 50 km/h, t_50m_; and average distance travelled from the time of starting the vehicle to achieving 50 km/h, S_50m_.

The last cycle performed for a dry asphalt surface, a wet asphalt surface (after rain), and a surface covered with a thin ice layer was aimed at designating the lateral acceleration acting on the tested vehicle while it was driven in a circle at maximum speed while maintaining stability. The test vehicle’s acceleration and speed were measured during the cycle. The plots of lateral acceleration acting on the vehicle after achieving its maximum speed during the cycle in given traffic conditions were developed. The following parameters were designated based on the conducted tests: minimum measured acceleration, a_cmin_; maximum measured acceleration, a_cmax_; the difference in accelerations, a_cmax_−a_cmin_; average measured acceleration, a_cm_; minimum measured speed while driving in a circle, V_cmin_; maximum measured speed while driving in a circle, V_cmax_; the difference in speed, V_cmax_−V_cmin_; and average speed while driving in a circle V_cm_.

### 2.2. Testing Instrumentation

The tests were conducted with the use of specialist measurement instrumentation intended for vehicle testing on the road. The instrumentation featured the TAA three-axial linear acceleration sensor, the Correvit S-350 Aqua longitudinal acceleration measurement sensor, and the uEEP-12 data acquisition station with the ARMS software for data analysis.

The TAA three-axial linear acceleration sensor enables three-axial dynamic acceleration measurement [45]. The sensor is adapted to measure acceleration in difficult industrial conditions and in a vehicle testing environment. The measurement signal is generated through a change in the sensor’s capacitive properties, caused by a change in speed. Then, the signal is converted into an electric signal which undergoes enhancement and filtering. The basic technical data of the TAA linear acceleration sensor is presented in Table 2.

The Correvit S-350 Aqua sensor enable accurate measurements of distance, longitudinal and lateral speeds, as well as angle in dynamic vehicle testing, e.g., during fixed motion in a circle according to ISO 4138 [46]. The sensor’s basic technical data is presented in Table 3. The Correvit S-350 Aqua sensor enables accurate recording of dynamic manoeuvres of different vehicles and on different surfaces [47].

The μEEP-12 system was used to acquire and assess the measurement data of the vehicle motion parameters during the road tests. The system is intended for mobile vehicle testing apps [48]. The μEEP-12 system interoperates with a laptop during the tests. The ARMS software enables the operation of the entire measurement system. The software allows for controlling the course of the tests. The μEEP-12 system enables acceleration, speed, and other parameters to be saved during dynamic vehicle manoeuvres, e.g., during emergency braking, rapid acceleration, and circular motion. The basic technical data of the μEEP-12 acquisition system is presented in Table 4.

## 3. Results and Discussion

The testing featured braking, acceleration, and circular motion cycles for a minibus vehicle on an asphalt surface covered with an invisible thin ice layer, and on a dry and wet asphalt surface. The wet surface represented the driving conditions after rain. The first stage of testing on each surface featured braking cycles. Ten braking cycles were performed on each surface. When analysing the test results, samples with a large error were discarded, e.g., incorrect recording of the measured quantities. Anon-parametric statistical Mann–Whitney U test was performed for a population of measured values on asphalt covered with a thin layer of ice in relation to dry asphalt and wet asphalt. A significance level of 0.05 was assumed. The tests were performed for a population of measured values: maximum recorded decelerations, a_max_; mean fully developed decelerations, MFDD; decelerations rise time, t_r_; braking distances, S_H_; maximum accelerations during acceleration cycles, a_rmax_; times between starting the car and achieving 50 km/h, t_50_; and distances travelled from the time of starting the car to achieving 50 km/h, S_50_. The calculated *p*-values were clearly less than the accepted level of significance. This means that there are statistically significant differences between the groups. Based on the test performed, it can be concluded that the data are from different populations.

**Figure 2:**
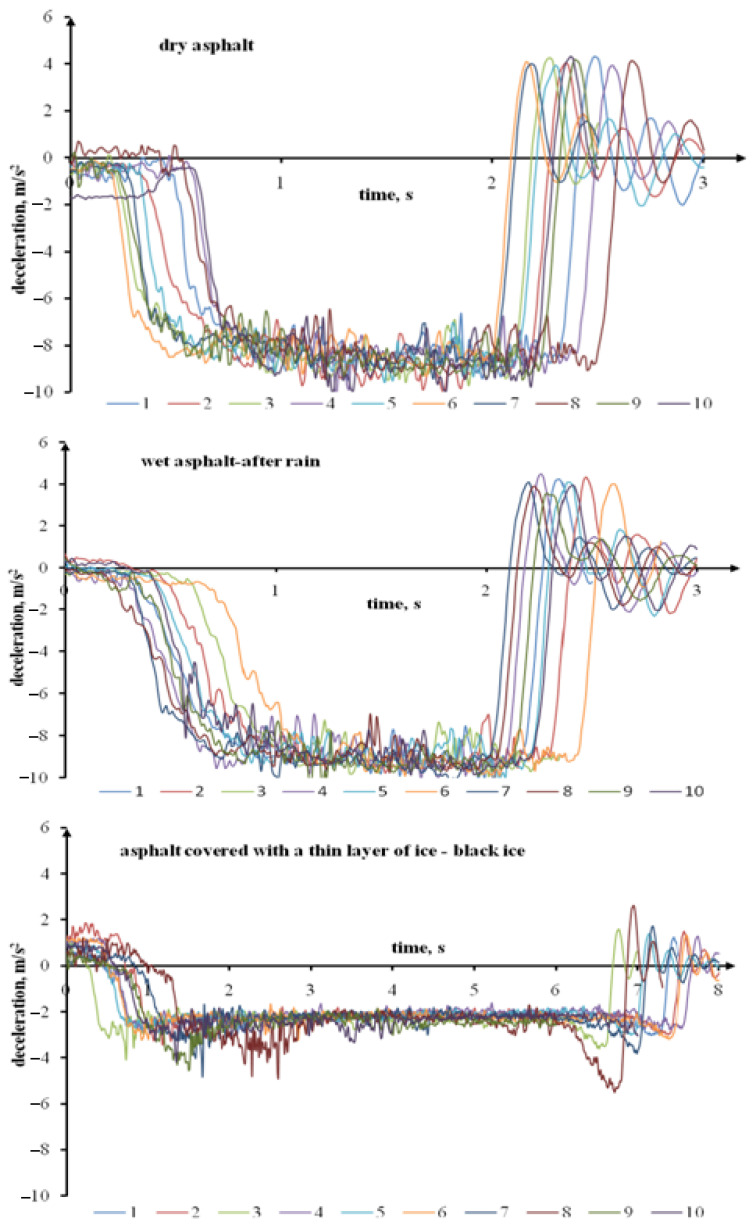
The Ford Transit’s deceleration during rapid braking on a dry surface, a wet (after rain) asphalt surface, and a surface covered with a thin ice layer with the initial speed of approximately 50 km/h.

Figure 2 presents the deceleration during rapid braking on a dry surface, a wet (after rain) asphalt surface, and a surface covered with a thin ice layer, for the initial braking speed of approximately 50 km/h. The plots demonstrate that the deceleration on a surface covered with an invisible thin ice layer deviates substantially from a dry or wet (after rain) asphalt surface. The deceleration on an icy surface is substantially lower. The braking takes a much longer time. On the other hand, the deceleration on a wet (after rain) asphalt does not differ substantially from the course of braking on a dry asphalt.

**Figure 3:**
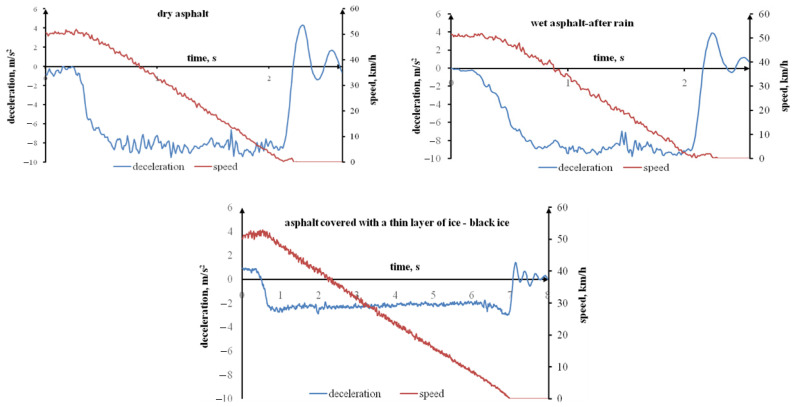
Examples of the Ford Transit’s deceleration and speed during rapid braking on a dry surface, a wet (after rain) asphalt surface, and a surface covered with a thin ice layer with the starting speed of approximately 50 km/h.

Plots of the test vehicle’s deceleration and speed changes were developed for each braking cycle. Figure 3 presents examples of the Ford Transit’s deceleration and speed during rapid braking on a dry surface, a wet (after rain) asphalt surface, and a surface covered with a thin ice layer with the starting speed of approximately 50 km/h. Selected motion parameters for the test vehicle, presented in Table 5 and Table 6, were designated based on these plots.

The initial braking speed, V_o_, maximum recorded deceleration, a_max_, and mean fully developed deceleration, MFDD, are presented in Table 5. The average values of the aforementioned parameters and the standard deviation were also calculated. The maximum deceleration, a_max_, and MFDD designated for the test vehicle on an asphalt surface covered with an invisible thin ice layer are substantially smaller than a_max_ and MFDD designated for dry and wet (after rain) asphalt. The average MFDD_m_ amounted to 2.27 m/s^2^ for icy asphalt and 8.21 m/s^2^ for dry asphalt. For a thick layer of undamaged black ice, which is difficult to see for an average driver, Martin and Schaefer stated the deceleration of 1.18–2.55 m/s^2^ [49]. Eddie, on an icy surface and for summer tyres, with the ABS enabled, achieved deceleration in the range of 1.28–1.96 m/s^2^. On the other hand, with the ABS disabled, the deceleration was in the range of 1.47–1.57 m/s^2^ [50]. For winter tyres, the same author achieved deceleration with the ABS enabled in the range of 1.47–2.65 m/s^2^, while with the ABS disabled 1.47–2.06 m/s^2^. The authors of this paper achieved higher MFDD_m_ by approximately 12.1% for wet asphalt when compared to dry asphalt. The wet asphalt did not feature any puddles or standing water. Such conditions reflected an asphalt surface after rain. For a loaded passenger car, when braking from 60 km/h and with the ABS enabled, the authors of paper [51] achieved an average deceleration of 5.25 m/s^2^ on dry asphalt and 5.41 m/s^2^ on wet asphalt. With the ABS disabled, the average deceleration amounted to 4.71 m/s^2^ on dry asphalt and 5.41 m/s^2^ on wet asphalt.

A wet (after rain) asphalt surface featuring no puddles or standing water did not negatively affect the braking effectiveness. It is possible to state that the grip coefficient did not decrease on a wet surface featuring no puddles or standing water. The braking effectiveness was slightly higher on this surface. This can be due to the enabled ABS system. Furthermore, the presence of water can result in a decreased temperature at the tyre-asphalt contact point, thereby reducing the grip coefficient. The braking effectiveness on dry asphalt could also have been affected by the presence of contaminants in the form of dust, invisible for the driver and researchers. The braking effectiveness on asphalt covered with a thin black ice layer was substantially worse when compared to dry asphalt and wet (after rain) asphalt. This substantially affects safety, especially when the driver is not aware that the asphalt is covered by a layer of black ice. This is confirmed by the braking times (t_r_, t_rm_, t_MFDD_, and t_MFDDm_) and braking distances (S_H_, S_Hm_) presented in Table 6. The average braking distance on asphalt covered with a black ice layer amounted to 46.44 m, whereas for dry asphalt 12.39 m, and wet (after rain) asphalt 11.06 m. The designated braking times on asphalt covered with an invisible ice layer are substantially greater than braking times on dry and wet (after rain) asphalt. The time t_MFDDm_ amounted to 4.58 s for icy asphalt, 1.22 s for dry asphalt, and 1.11 s for wet (after rain) asphalt. It is very probable that a driver who is unaware that he or she is driving on an icy road, who does not maintain adequate speed and distance from the car in front, will cause a collision. Works are in progress on systems that recognize surface slipperiness and notify the driver [35,36]. The driver should reduce the speed and increase the distance from the car in front.

The rapid braking cycles were performed after reducing the initial speed from approximately 50 km/h to approximately 30 km/h. The values of parameters V_o_, V_om_, a_max_, a_maxm_, MFDD, and MFDD_m_ are presented in Table 7. The average maximum deceleration, a_maxm_, measured on icy asphalt amounted to 4.35 m/s^2^ and is substantially lower than on dry asphalt 10.90 m/s^2^, and wet (after rain) asphalt 10.08 m/s^2^. The average mean fully developed deceleration, MFDD_m_, on a surface covered with ice amounted to 2.87 m/s^2^ and is substantially lower than the value achieved on dry asphalt 8.85 m/s^2^, and wet (after rain) asphalt 8.28 m/s^2^. MFDD_m_ for icy asphalt is slightly higher for the initial speed of approximately 30 km/h when compared to the value measured for the initial speed of approximately 50 km/h. This could have resulted from the fact that the cycles at V_o_ of approximately 30 km/h were performed right after the cycles at V_o_ of approximately 50 km/h. The ice layer covering the asphalt surface was thin and became damaged during subsequent cycles. For a thin black ice layer which was difficult to see for the driver and partially damaged by locked sliding tyres, Martin and Schaefer achieved deceleration in the range of 1.67–4.81 m/s^2^ [49].

Table 8 presents the braking times and braking distances for a Ford Transit during rapid braking on a dry surface, a wet (after rain) surface, and an icy asphalt with the initial speed of approximately 30 km/h. The braking times on asphalt covered with an invisible ice layer were slightly lower when compared to the times measured on dry and wet (after rain) asphalt. The average braking distance amounted to 12.94 m for icy asphalt, 4.68 m for dry asphalt, and 4.93 m for wet (after rain) asphalt. The braking distance was over 2.5 times longer for icy asphalt than for dry and wet (after rain) asphalt. The average braking distance amounted to 46.44 m on invisible ice with the initial speed of approximately 50 km/h and 12.94 m with the initial speed of approximately 30 km/h. When reducing the initial speed by approximately 20 km/h, the braking distance is reduced 3.6 times. The above analysis demonstrates the importance of the road surface and whether the driver is able to assess it and adapt his or her driving speed in terms of safety.

**Figure 4:**
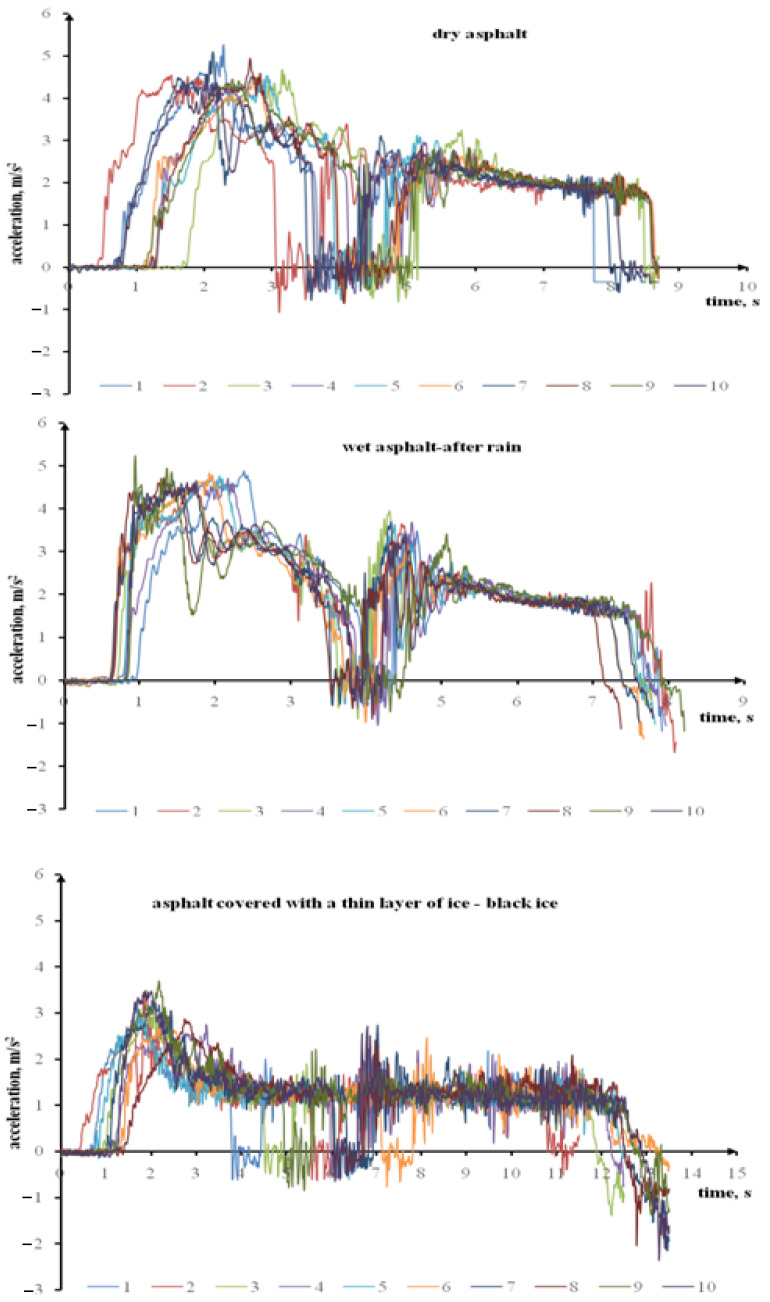
The Ford Transit’s longitudinal acceleration during rapid acceleration on a dry surface, a wet (after rain) asphalt surface, and an asphalt surface covered with a thin layer of ice.

The braking manoeuvre is most important in terms of road traffic safety. The acceleration manoeuvre is also important. A driver who enters an icy road can be a hazard for other vehicles as he or she will not accelerate his or her car sufficiently quickly. The testing included rapid acceleration cycles performed with a Ford Transit adapted for passenger transport. Figure 4 presents the Ford Transit’s longitudinal acceleration during rapid acceleration on a dry surface, a wet (after rain) asphalt surface, and an asphalt surface covered with a thin layer of ice up to 50 km/h. The plot waveforms demonstrate that the maximum acceleration on an icy road is lower when compared to dry and wet (after rain) surfaces. The time taken to achieve the assumed speed (acceleration) is also longer. The acceleration waveforms for wet (after rain) asphalt demonstrate that the vehicle achieved maximum acceleration quicker.

**Figure 5:**
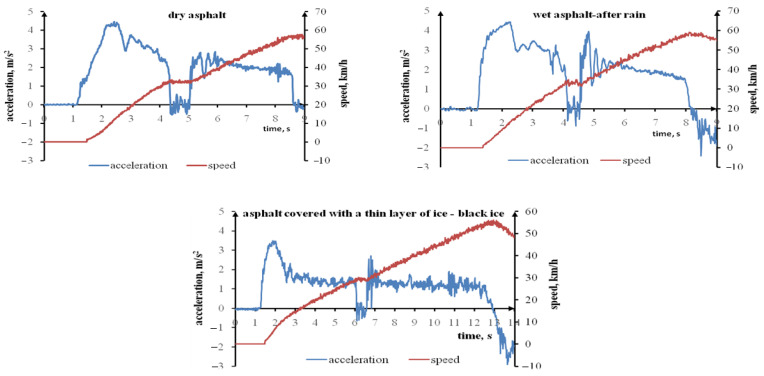
An example of the Ford Transit’s acceleration and speed during rapid acceleration on a dry surface, a wet (after rain) asphalt surface, and an asphalt surface covered with a thin layer of ice.

Figure 5 presents examples of longitudinal acceleration and longitudinal speed for the tested vehicle during rapid acceleration on a dry, a wet (after rain), and an icy asphalt surface. The following vehicle parameters were designated based on these plots: maximum acceleration during acceleration cycles, a_rmax_; time between starting the vehicle and achieving maximum acceleration, t_armax_; time between starting the vehicle and achieving 50 km/h, t_50_; and distance travelled from the time of starting the vehicle to achieving 50 km/h, S_50_. The values of the aforementioned parameters for particular acceleration cycles, their average values, and the standard deviations are presented in Table 9. The distance required to accelerate the tested vehicle to 50 km/h on icy asphalt amounted to 81.68 m. The distance on dry asphalt amounted to 47.36 m, while on wet (after rain) asphalt 43.93 m. The clearly lower grip on the asphalt surface covered with a thin ice layer causes the acceleration to the set speed to be slower and requires travelling a longer distance. In critical situations, this can substantially affect road traffic safety.

The average maximum longitudinal acceleration for the rapid acceleration cycles performed by the tested vehicle on asphalt covered with a thin ice layer amounted to 3.18 m/s^2^. On dry asphalt, this parameter amounted to 4.71 m/s^2^, while on wet (after rain) asphalt 5.50 m/s^2^. This is yet another proof that covering asphalt with water can increase the tyre grip. The asphalt featured no puddles or standing water. Covering the asphalt with water could have cleared the dust which affect the tyre grip.

Navin et al. specified the measured acceleration on smooth ice as 0.78 m/s^2^ [52]. The tested vehicle’s average maximum acceleration during acceleration cycles was relatively high (3.18 m/s^2^). This most probably resulted from the fact that the tyres damaged the invisible thin black ice layer rather quickly during acceleration. The measured acceleration increased in such a case. Furthermore, the tested vehicle was equipped with the ASR traction control system intended to prevent the tyres from sliding during start-up and acceleration.

**Figure 6:**
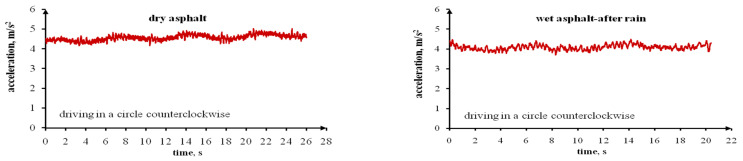

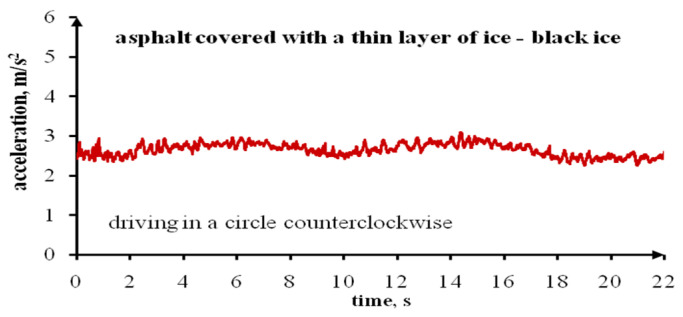
The Ford Transit’s lateral accelerations when driving in a circle counterclockwise (to the left), with the highest possible speed on a dry, wet (after rain) and icy asphalt surface.

**Figure 7:**
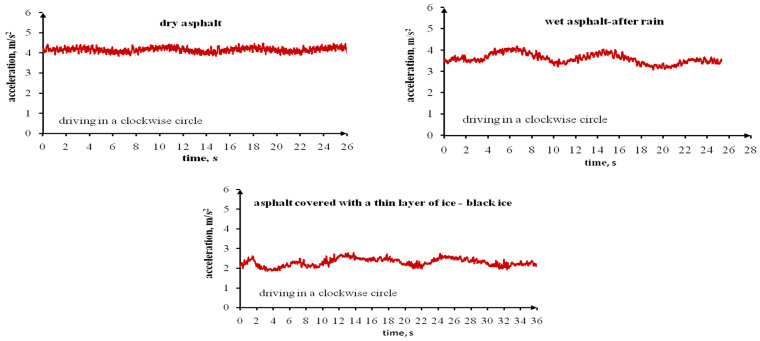
The Ford Transit’s lateral accelerations when driving in a circle clockwise (to the right), with the highest possible speed on a dry, wet (after rain) and icy asphalt surface.

The last stage of each test series on dry, wet (after rain) and icy asphalt featured cycles of driving in a circle with maximum speed while maintaining stability. Driving in an arch at excessive speeds can result in stability loss and even in overturning the vehicle, especially a vehicle with a high centre of gravity. This is the result of lateral accelerations that affect the vehicle. The values of permissible lateral accelerations affecting a vehicle driven in an arch also depend on the road surface, i.e., the tyre grip. Figure 6 presents the lateral accelerations affecting the tested vehicle when driven in a circle, counter clockwise, on dry, wet (after rain) and icy asphalt. On the other hand, Figure 7 presents the lateral accelerations affecting the tested vehicle when driven in a circle, clockwise. These plots were used to read the specific values of lateral accelerations and speeds of the tested vehicle when driven in a circle. These values are presented in Table 10. The designated lateral accelerations and speeds are higher when driving in a circle counter clockwise (to the left) when compared to driving in a circle clockwise (to the right). This applies to all asphalt surfaces. The lateral accelerations affecting the tested vehicle are smaller for asphalt covered with a thin ice layer, greater for wet (after rain) asphalt and highest for dry asphalt. The average lateral acceleration for icy asphalt amounted to 2.65 m/s^2^ (driving to the left) and 2.30 m/s^2^ (driving to the right), while for dry asphalt: 4.56 m/s^2^ and 4.16 m/s^2^, respectively, and for wet (after rain) asphalt: 4.08 m/s^2^ and 3.60 m/s^2^, respectively. The ice layer was thin and practically invisible. When driving in a circle, the tyres damaged the ice layer, and this affected the lateral accelerations affecting the vehicle. With a thick ice layer, these accelerations were undoubtedly smaller. The speeds measured for the Ford Transit when driven in a circle were lower on icy asphalt and higher on dry asphalt. The vehicle speed on wet (after rain) asphalt was slightly lower than on dry asphalt. It is obvious that when driving in a circle on icy asphalt, a driver must maintain a lower speed. It is difficult to determine this value. It depends on the ice layer’s thickness and the possibility of it being cracked and damaged by tyres, ambient temperatures, and other factors. To improve the safety, it is possible to equip road vehicles with a system that will notify the driver that the road is covered with ice and requires a lowering of the driving speed.

The fact that the asphalt is wet does not necessarily mean that the tyre grip is lower. The important aspect is the definition of the term “wet asphalt”. In this paper, wet asphalt was asphalt after rain (after being covered with water), without puddles or standing water. Higher deceleration from 50 km/h and higher acceleration during acceleration cycles were achieved on this asphalt. It was possible to achieve greater tyre grip when braking or accelerating on wet asphalt due to the lower temperatures caused by the cooling of the tyre-asphalt contact point by water. In addition, dry asphalt could have been covered with dust, which also could have affected the grip. Invisible contaminants could have been removed from the asphalt when it was covered with water. The conducted tests demonstrate the many factors that affect common driving manoeuvres. This makes the analysis of road accidents difficult and ambiguous. The variation of factors than can affect safe driving means that drivers should have the ability to assess the road conditions and take them into account when making decisions on the road. The life and health of the driver and other road users depend on it.

The analysis conducted in this paper is important for drivers, especially drivers with little experience. Drivers should be made aware of the impact of surface condition on braking manoeuvres. The case of the so-called black ice on an asphalt surface discussed in the tests is especially dangerous, as it can be invisible for the driver. The driver must be aware that the road surface can be covered with ice at ambient temperatures around and below zero degrees. This will depend on atmospheric conditions and road location, e.g., a forest road or a road near water reservoirs. A good solution to this issue would be to fit vehicles with a system that will notify the driver about occurring hazards deriving from a slippery road surface. This may be the subject of further studies conducted by this paper’s authors. Theofilatos and Yannis stated that it is necessary to intensify studies on the impact of weather on road traffic safety with the use of real-time data [53]. The tests presented in this paper provide information about the behaviour of a selected vehicle type in critical road traffic conditions. This information should be provided to the driver in the form of warning signals on road slipperiness and the need to reduce speed.

## 4. Conclusions

The testing of parameters during braking, acceleration, and driving in a circle was conducted for relatively rarely tested road conditions. The measurements were conducted on an asphalt surface covered with an invisible thin ice layer. It is referred to as black ice. It can be a huge surprise for the driver as he or she may simply not see it. The Ford Transit’s motion parameters measured on asphalt covered with a thin ice layer were compared with the same parameters measured on dry and wet asphalt. The wet asphalt reflected the road conditions after rain. The asphalt featured no puddles or standing water. Based on the conducted testing, it is possible to make the following conclusions concerning the designated parameters:

- Parameter a_maxm_ for rapid braking cycles, measured using the acceleration sensor, for V_o_ of approximately 50 km/h, on asphalt covered with a thin ice layer was 2.6 times lower than on dry asphalt and 2.7 times lower than for wet (after rain) asphalt. On the other hand, for V_o_ of approximately30 km/h, parameter a_maxm_ was 2.5 times lower than on dry asphalt and 2.3 times lower than on wet (after rain) asphalt.
- Parameter MFDD_m_, calculated based on the tested vehicle’s measured speed, for V_o_ of approximately 50 km/h, on asphalt covered with a thin ice layer was 3.6 times lower than on dry asphalt and 4.1 times lower than on wet (after rain) asphalt. For V_o_ of approximately 30 km/h, parameter MFDD_m_ was 3.1 times lower than on dry asphalt and 2.9 times lower than on wet (after rain) asphalt.
- The braking times t_rm_ and t_MFDDm_ for V_o_ of approximately 50 km/h on asphalt covered with a thin ice layer were 2.8 and 3.8 times higher, respectively, than on dry asphalt, and 2.3 and 4.1 times higher, respectively, than on wet (after rain) asphalt. For V_o_ of approximately 50 km/h, the braking times t_rm_ and t_MFDDm_ on asphalt covered with a thin ice layer were 2.3 and 2.9 times higher, respectively, than on dry asphalt, and 1.7 and 2.8 times higher, respectively, than on wet (after rain) asphalt.
- The average braking distance S_Hm_ for V_o_ of approximately 50 km/h on asphalt covered with a thin ice layer was 3.7 times greater than on dry asphalt and 4.2 times greater than on wet (after rain) asphalt. Following the reduction of V_o_ to approximately 30 km/h, the braking distance on icy asphalt was 2.8 and 2.6 times greater than on dry and wet (after rain) asphalt, respectively.
- The average maximum acceleration, a_rmaxm_, during acceleration cycles on icy asphalt was lower by 32.48% than on dry asphalt and by 42.18% than on wet (after rain) asphalt.
- The average time required to achieve 50 km/h, t_50m_, on asphalt covered with a thin ice layer was higher by 72.93% than on dry asphalt and by 90.88% than on wet (after rain) asphalt.
- The distance travelled before achieving 50 km/h, S_50m_, on asphalt covered with a thin ice layer was higher by 72.47% than on dry asphalt and by 85.93% than on wet (after rain) asphalt.
- The average lateral acceleration, a_cm_, when driving in a circle with the maximum possible speed while maintaining stability on asphalt covered with a thin ice layer, counter clockwise, was lower by 41.89% than on dry asphalt and by 35.05% than on wet (after rain) asphalt. On the other hand, the average lateral acceleration when driving a circle clockwise on asphalt covered with a thin ice layer was lower by 44.71% and 36.11% than on dry and wet (after rain) asphalt, respectively.
- The average speed, V_cm,_ when driving in a circle counter clockwise on asphalt covered with a thin ice layer was lower by 19.02% and 17.33% than on dry and wet (after rain) asphalt, respectively. On the other hand, when driving in a circle clockwise, the average speed, V_cm_, on icy asphalt was lower by 31.48% and 24.92% than on dry and wet (after rain) asphalt, respectively.

The testing concerned braking, acceleration manoeuvres, and driving in a circle with a vehicle that can transport up to nine people. It is referred to as a minibus. The vehicle has great height and a higher centre of gravity when compared to typical passenger cars. It is necessary to take these factors into consideration when driving such a vehicle, especially in difficult road conditions, e.g., on an icy road. The results obtained in the tests can be used to simulate the motion of minibus-type vehicles in critical conditions and when testing the causes of road accidents featuring such vehicles. The test results obtained cannot be generalised to all vehicles of this type. There are very different designs in the minibus group of vehicles. The tests were carried out for a selected design with specific equipment. The parameters of the braking process depend, among other things, on the design of the braking system, the type of tyres used, the size and weight of the vehicle, the position of the centre of gravity, the systems used to control the vehicle and protect it against skidding and wheel lock, and other systems supporting the driver. It would be advisable to carry out such tests for different designs of minibus vehicles and with different equipment.

## Figures and Tables

**Table 1:** Tyre parameters of the Ford Transit test vehicle.

Parameter	Unit	Value
Width	mm	205
Profile	%	65
Diameter	cal	16
Load index	-	107/105
Speed index	-	T
Maximum speed	km/h	190

**Table 2:** Basic technical data of the TAA linear acceleration sensor [45].

Performance Specifications		Unit	Value
Measurement range		g	±3
Operating temperature range		°C	–40 … 85
power supply		V	7 … 42
Limit frequency		Hz	5
Sensitivity	Nominal value	mV/g	666
	Tolerance	%FSO	±1
Non-linearity, nominal value		%	±0.2
Transverse sensitivity, nominal value		%FSO	±2
Zero rate bias drift (–40 … 85 °C max.)		g	0.2

**Table 3:** Basic technical data of the Correvit S-350 Aqua optoelectrical sensor [47].

Performance Specifications	Unit	Value
Speed range	km/h	0.5 … 250
Distance resolution	mm	2.47
Measurement accuracy	%FSO	<±0.2
Angle range	°	±40
Angle resolution	°	<±0.1
Meas. Accuracy angle	°	<±0.2
Measurement frequency	Hz	250
Working distance/range	mm	350 ± 100

**Table 4:** Basic technical data of the μEEP-12 data acquisition system [48].

Performance Specifications	Unit	Value
Number of channels	-	16
Input voltage range (adjustable)	mV … V	50 … 60
Sampling rate per channel max.	kHz	50
Input impedance	GΩ	>1
Linearity	%	<0.05
Zero offset drift	LSB	2
Bandwidth (various filters adjustable)	kHz	8

**Table 5:** The Ford Transit’s deceleration during rapid braking on a dry surface, a wet (after rain) asphalt surface, and a surface covered with a thin ice layer with the initial speed of approximately 50 km/h.

Asphalt	Test Run No.	V_o_ km/h	V_om_ km/h	SD	a_max_ m/s^2^	a_maxm_ m/s^2^	SD	MFDD m/s^2^	MFDD_m_ m/s^2^	SD
dry	1	50.89	51.27	2.71	9.48	9.66	0.34	8.47	8.21	0.21
	2	51.82			10.12			8.43		
	3	53.23			9.70			8.14		
	4	51.89			10.26			8.42		
	5	51.18			9.56			7.90		
	6	51.16			9.31			8.05		
	7	47.62			9.40			8.30		
	8	54.04			9.42			7.95		
	9	54.82			9.36			8.32		
	10	46.00			10.00			8.12		
wet-after rain	1	52.96	51.29	1.28	10.18	10.06	0.25	8.81	9.20	0.33
	2	51.77			9.99			8.96		
	3	51.11			9.94			9.21		
	4	50.20			10.23			8.88		
	5	49.35			9.90			9.67		
	6	50.67			10.09			9.50		
	7	50.69			9.76			9.60		
	8	51.52			10.59			8.93		
	9	53.38			9.84			9.25		
black ice	1	52.18	52.24	0.94	3.02	3.75	0.76	2.17	2.27	0.11
	2	52.67			3.44			2.25		
	3	51.11			4.08			2.19		
	4	53.79			2.89			2.16		
	5	52.57			3.01			2.25		
	6	51.84			4.82			2.28		
	7	50.94			4.55			2.46		
	8	52.82			4.18			2.43		

**Table 6:** The Ford Transit’s deceleration times and braking distances during rapid braking on a dry surface, a wet (after rain) asphalt surface, and a surface covered with a thin ice layer with the initial speed of approximately 50 km/h.

Asphalt	Test Run No.	V_o_ km/h	t_r_ s	t_rm_ s	SD	t_MFDD_ s	t_MFDDm_ s	SD	S_H_ m	S_Hm_ m	SD
dry	1	50.89	0.53	0.48	0.06	1.21	1.22	0.07	11.79	12.39	1.33
	2	51.82	0.52			1.19			12.29		
	3	53.23	0.49			1.26			13.42		
	4	51.89	0.45			1.19			12.34		
	5	51.18	0.39			1.23			12.80		
	6	51.16	0.47			1.25			12.55		
	7	47.62	0.51			1.14			10.54		
	8	54.04	0.48			1.32			14.16		
	9	54.82	0.58			1.29			13.93		
	10	46.00	0.41			1.07			10.05		
wet-after rain	1	52.96	0.55	0.58	0.05	1.17	1.11	0.04	12.28	11.06	0.82
	2	51.77	0.60			1.13			11.53		
	3	51.11	0.53			1.10			10.94		
	4	50.20	0.63			1.10			10.95		
	5	49.35	0.61			1.05			9.72		
	6	50.67	0.57			1.07			10.42		
	7	50.69	0.66			1.07			10.32		
	8	51.52	0.54			1.16			11.46		
	9	53.38	0.53			1.15			11.88		
black ice	1	52.18	1.35	1.35	0.14	4.81	4.58	0.28	48.36	46.44	3.23
	2	52.67	1.60			4.69			47.63		
	3	51.11	1.38			4.52			46.05		
	4	53.79	1.39			4.98			51.69		
	5	52.57	1.29			4.68			47.43		
	6	51.84	1.20			4.47			45.42		
	7	50.94	1.13			4.05			40.66		
	8	52.82	1.42			4.47			44.28		

**Table 7:** The Ford Transit’s deceleration during rapid braking on a dry surface, a wet (after rain) asphalt surface, and a surface covered with a thin ice layer with the initial speed of approximately 30 km/h.

Asphalt	Test Run No.	V_o_ km/h	V_om_ km/h	SD	a_max_ m/s^2^	a_maxm_ m/s^2^	SD	MFDD m/s^2^	MFDD_m_ m/s^2^	SD
dry	1	30.65	32.72	1.09	10.98	10.90	0.22	8.84	8.85	0.36
	2	32.41			11.00			9.12		
	3	33.43			10.74			8.51		
	4	33.48			10.89			8.37		
	5	33.70			10.86			9.09		
	6	31.92			11.31			8.68		
	7	32.43			10.68			8.94		
	8	34.53			10.67			8.85		
	9	32.33			11.18			9.59		
	10	32.36			10.73			8.51		
wet-after rain	1	32.97	32.51	1.01	9.65	10.08	0.31	8.71	8.28	0.35
	2	32.77			10.05			8.15		
	3	33.58			9.86			8.34		
	4	33.31			9.74			8.00		
	5	32.94			10.40			8.25		
	6	33.36			10.03			8.94		
	7	31.11			10.14			7.82		
	8	31.70			10.64			8.23		
	9	30.89			10.19			8.09		
black ice	1	30.43	30.79	1.29	3.81	4.35	0.67	2.84	2.87	0.44
	2	30.96			5.23			3.48		
	3	30.72			5.01			3.33		
	4	29.28			3.25			2.63		
	5	33.11			4.46			3.62		
	6	31.99			5.28			2.80		
	7	30.45			4.32			2.47		
	8	28.52			3.94			2.48		
	9	31.16			4.39			2.45		
	10	31.23			3.82			2.59		

**Table 8:** The Ford Transit’s deceleration times and braking distances during rapid braking on a dry surface, a wet (after rain) asphalt surface, and a surface covered with a thin ice layer with the initial speed of approximately 30 km/h.

Asphalt	Test Run No.	V_o_ km/h	t_r_ s	t_rm_ s	SD	t_MFDD_ s	t_MFDDm_ s	SD	S_H_ m	S_Hm_ m	SD
dry	1	30.65	0.38	0.35	0.03	0.66	0.72	0.03	4.10	4.68	0.39
	2	32.41	0.34			0.70			4.44		
	3	33.43	0.33			0.74			5.07		
	4	33.48	0.38			0.76			5.17		
	5	33.70	0.36			0.72			4.82		
	6	31.92	0.31			0.71			4.53		
	7	32.43	0.37			0.71			4.54		
	8	34.53	0.34			0.75			5.20		
	9	32.33	0.39			0.67			4.20		
	10	32.36	0.31			0.73			4.75		
wet-after rain	1	32.97	0.62	0.46	0.08	0.73	0.75	0.03	4.81	4.93	0.26
	2	32.77	0.52			0.78			5.08		
	3	33.58	0.43			0.78			5.21		
	4	33.31	0.38			0.80			5.35		
	5	32.94	0.43			0.76			5.07		
	6	33.36	0.51			0.74			4.80		
	7	31.11	0.43			0.74			4.77		
	8	31.70	0.37			0.72			4.71		
	9	30.89	0.45			0.73			4.55		
black ice	1	30.43	0.75	0.79	0.08	2.15	2.09	0.25	12.60	12.94	1.61
	2	30.96	0.81			1.75			10.62		
	3	30.72	0.84			1.75			10.92		
	4	29.28	0.95			2.23			12.57		
	5	33.11	0.73			1.75			11.68		
	6	31.99	0.75			2.10			14.08		
	7	30.45	0.79			2.32			14.50		
	8	28.52	0.83			2.19			12.64		
	9	31.16	0.77			2.40			15.30		
	10	31.23	0.67			2.30			14.52		

**Table 9:** The Ford Transit’s selected motion parameters during rapid acceleration on a dry surface, a wet (after rain) asphalt, and asphalt covered with a thin ice layer.

Asphalt	Test Run No.	a_rmax_ m/s^2^	a_rmaxm_ m/s^2^	SD	t_armax_ s	t_armaxm_ s	SD	t_50_ s	t_50m_ s	SD	S_50_ m	S_50m_ m	SD
dry	1	5.25	4.71	0.29	1.60	1.42	0.18	6.29	6.17	0.18	48.18	47.36	1.43
	2	4.54			1.11			5.88			44.92		
	3	4.68			1.42			5.86			45.33		
	4	4.53			1.46			6.12			47.41		
	5	4.53			1.66			6.11			46.40		
	6	4.47			1.63			6.39			49.17		
	7	5.10			1.39			6.22			47.60		
	8	4.94			1.39			6.30			49.08		
	9	4.45			1.27			6,31			47.81		
	10	4.57			1.26			6.26			47.70		
wet-after rain	1	4.88	5.50	0.22	1.43	1.02	0.40	5.53	5.59	0.16	43.11	43.93	1.50
	2	4.52			1.06			5.52			43.15		
	3	4.43			1.01			5.41			41.70		
	4	4.70			1.48			5.66			43.12		
	5	4.77			1.27			5.40			43.45		
	6	4.84			1.32			5.57			44.34		
	7	4.64			0.86			5.56			43.57		
	8	4.72			0.71			5.67			45.14		
	9	5.23			0.12			5.97			47.25		
	10	4.65			0.95			5.56			44.44		
black ice	1	3.26	3.18	0.29	0.96	1.04	0.24	10.34	10.67	0.57	73.08	81.68	4.79
	2	3.44			1.47			11.50			86.91		
	3	2.98			1.15			11.06			83.32		
	4	2.94			1.14			10.80			84.51		
	5	2.90			0.87			11.24			85.49		
	6	2.97			0.96			11.26			88.18		
	7	3.26			0.92			10.12			80.25		
	8	2.87			1.36			10.36			77.51		
	9	3.70			0.92			9.91			77.92		
	10	3.48			0.65			10.12			79.59		

**Table 10:** The Ford Transit’s lateral accelerations and speeds when driving in a circle with the highest possible speed on a dry, wet (after rain) and icy asphalt surface.

Parameter	asphalt					
	Dry		Wet-After Rain		Black Ice	
	Driving Direction		Driving Direction		Driving Direction	
	Left	Right	Left	Right	Left	Right
Minimum measured acceleration a_cmin_, m/s^2^	4.16	3.83	3.70	3.07	2.25	1,85
Maximum measured acceleration a_cmax_, m/s^2^	5.02	4.48	4.48	4.18	3.08	2.79
a_cmin_−a_cmin_, m/s^2^	1.06	0.65	0.78	1.12	0.83	0.94
Mean measured acceleration a_cm_, m/s^2^	4.56	4.16	4.08	3.60	2.65	2.30
Minimum measured speed V_cmin_, km/h	15.00	15.05	14.75	13.36	12.24	9.43
Maximum measured speed V_cmax_, km/h	18.20	17.49	18.05	16.56	14.83	12.90
V_cmax_−V_cmin_, km/h	3.20	2.44	3.30	3.20	2.59	3.47
Mean measured speed V_cm_, km/h	16.67	16.23	16.33	14.81	13.50	11.12

## Data Availability

The data presented in this study are available from the corresponding author on reasonable request.
